# Recent advances in understanding the roles of T cells in atrial fibrillation

**DOI:** 10.1038/s44325-024-00026-6

**Published:** 2024-09-30

**Authors:** Jiu Pu, Yimei Du

**Affiliations:** 1 Department of Cardiology, Union Hospital, Tongji Medical College, Huazhong University of Science and Technology, Wuhan, China; 2 Research Center of Ion Channelopathy, Union Hospital, Tongji Medical College, Huazhong University of Science and Technology, Wuhan, China; 3 Institute of Cardiology, Union Hospital, Tongji Medical College, Huazhong University of Science and Technology, Wuhan, China; 4 Key Lab for Biological Targeted Therapy of Education Ministry and Hubei Province, Union Hospital, Tongji Medical College, Huazhong University of Science and Technology, Wuhan, China; 5 Hubei Provincial Engineering Research Center of Immunological Diagnosis and Therapy for Cardiovascular Diseases, Union Hospital, Tongji Medical College, Huazhong University of Science and Technology, Wuhan, China

**Keywords:** Immunological disorders, Inflammation, Cardiovascular biology

## Abstract

Atrial fibrillation (AF) is a common arrhythmia associated with severe outcomes like heart failure and stroke. Recent studies highlight the crucial role of T in AF. Clinical studies have observed elevated levels of CD4^+^CD28^null^ T cells, Th17/Treg cells, CD8^+^ cells, and related markers in the peripheral blood or atrial tissue of AF patients, correlating with disease severity and cardiovascular events. These T cell subsets contribute to AF through: (1) releasing inflammatory factors like TNF-α and IL-17 which affect calcium homeostasis and electrical activity in atrial myocytes and/or promote atrial fibrosis; (2) recruiting inflammatory cells such as macrophages, causing local inflammation, oxidative stress, and atrial remodeling; (3) secreting cytotoxic proteins like perforin and granzymes, inducing apoptosis in atrial myocytes and affecting their action potentials; (4) direct contact, influencing atrial myocyte electrophysiology. Understanding these T cell-mediated mechanisms may uncover new therapeutic targets for AF.

## Introduction

**Fig. 1:**
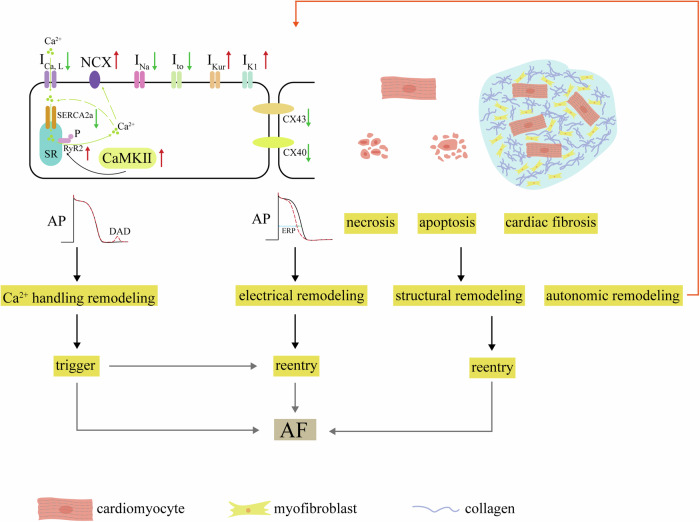
Overview of pathogenesis of AF. Decreasing I_Ca,L_ and SERCA2a along with increasing CaMKII, NCX (Na^+^/Ca^2+^-exchanger) and RyR2 expression and phosphorylation result in DADs, known as Ca^2+^ handling abnormities. Trigger is from Ca^2+^ handling abnormities. Ion channel dysfunctions including decreasing I_Na_ and I_to_, increasing I_Kur_ and I_K1_, decreasing and misdistribution of connexin-40 and connexin-43 cause electrical remodeling, known as decreasing ERP. Structural remodeling is due to necrosis and apoptosis of cardiomyocytes and cardiac fibrosis. Both electrical and structural remodeling can induce reentry. Trigger and reentry promote AF occurrence.

Atrial fibrillation (AF) stands out as one of the most prevalent clinical arrhythmias, significantly impacting the health of millions worldwide^1^. Individuals with AF face a markedly increased risk of severe consequences, including heart failure, stroke, and cardiovascular mortality. This heightened risk places a substantial burden on both the quality of life for affected individuals and the broader public health system^2,3^. Despite years of research into the etiology and mechanisms of AF, the precise underlying principles remain incompletely elucidated^4^. Previous studies suggest that the occurrence of AF is intricately linked to multiple factors, including electrical remodeling, structural remodeling, abnormalities in intracellular Ca^2+^ handling, and autonomic remodeling^5,6^. These factors interact in a complex pathophysiological process. A general overview is provided here (Fig. 1); for more information, see detailed reviews^7,8^. Electrical remodeling, a hallmark of AF, is characterized by significant modifications in the refractory periods and conduction velocity of atrial cardiomyocytes. These alterations are not only driven by sustained high atrial rates but are also profoundly influenced by various pathological conditions, such as heart failure^9^ and obesity^10^. At the molecular level, this remodeling process is closely associated with pronounced changes in ion channel currents, particularly reductions in transient outward potassium current (I_to_), L-type calcium current (I_Ca,L_), and sodium current (I_Na_), as well as disturbances in connexin expression and distribution. Structural remodeling of the atria involves cardiomyocyte apoptosis/necrosis, atrial fibrosis, and deposition of interstitial collagen. Ca^2+^ plays a pivotal role in the excitation-contraction coupling of cardiac muscle. When an action potential occurs, membrane depolarization triggers the opening of I_Ca,L_, allowing Ca^2+^ to enter. This Ca^2+^ influx then prompts the release of Ca^2+^ from the sarcoplasmic reticulum (SR) via ryanodine receptors (RyR2), generating calcium sparks and rapidly increasing intracellular calcium concentration ([Ca^2+^]_i_), known as calcium transient, leading to myocardial contraction. Later, Ca^2+^ is reabsorbed into the SR by the calcium pump (SERCA2a) and expelled from the cell via sodium-calcium exchange (NCX), aiding myocardial relaxation. Any disruption in these regulatory factors can disturb this delicate balance, leading to calcium homeostasis imbalance. To enhance understanding of the pathological foundation underlying the development of AF, the European Heart Rhythm Association (EHRA), the Heart Rhythm Society (HRS), the Asia Pacific Heart Rhythm Society (APHRS), and the Latin American Heart Rhythm Society (LAHRS) recently released an expert consensus on atrial cardiomyopathy (ACM)^11^. ACM is defined as any complex of structural, architectural, contractile or electrophysiological changes affecting the atria with the potential to produce clinically-relevant manifestations. ACM increases the risk of AF, and AF may, in turn, accelerate the progression of ACM.

Recent research has identified a novel factor contributing to AF—immune system modulation^12,13^. While prior reviews have extensively discussed the role of innate immune cells in AF, particularly how macrophages release inflammatory cytokines that promote fibrosis^14–16^, emerging evidence also highlights the critical role of the NLRP3 inflammasome. The activation of the NLRP3 inflammasome involves “priming” and “triggering” of a complex consisting of NLRP3, ASC, and pro-caspase-1, leading to the activation of caspase-1. Activated caspase-1 then facilitates the maturation and release of pro-inflammatory cytokines IL-1β and IL-18 through gasdermin-D-mediated membrane pores. Furthermore, in cardiomyocytes, NLRP3 activation disrupts Ca^2+^ handling by phosphorylating RyR2 and phospholamban, likely through enhanced CaMKII pathways and impaired AMPK signaling^17–19^. This article aims to elucidate the involvement of acquired immune cells in AF, expanding our understanding of the condition beyond the innate immune mechanisms.

## Overview of T cells

**Fig. 2:**
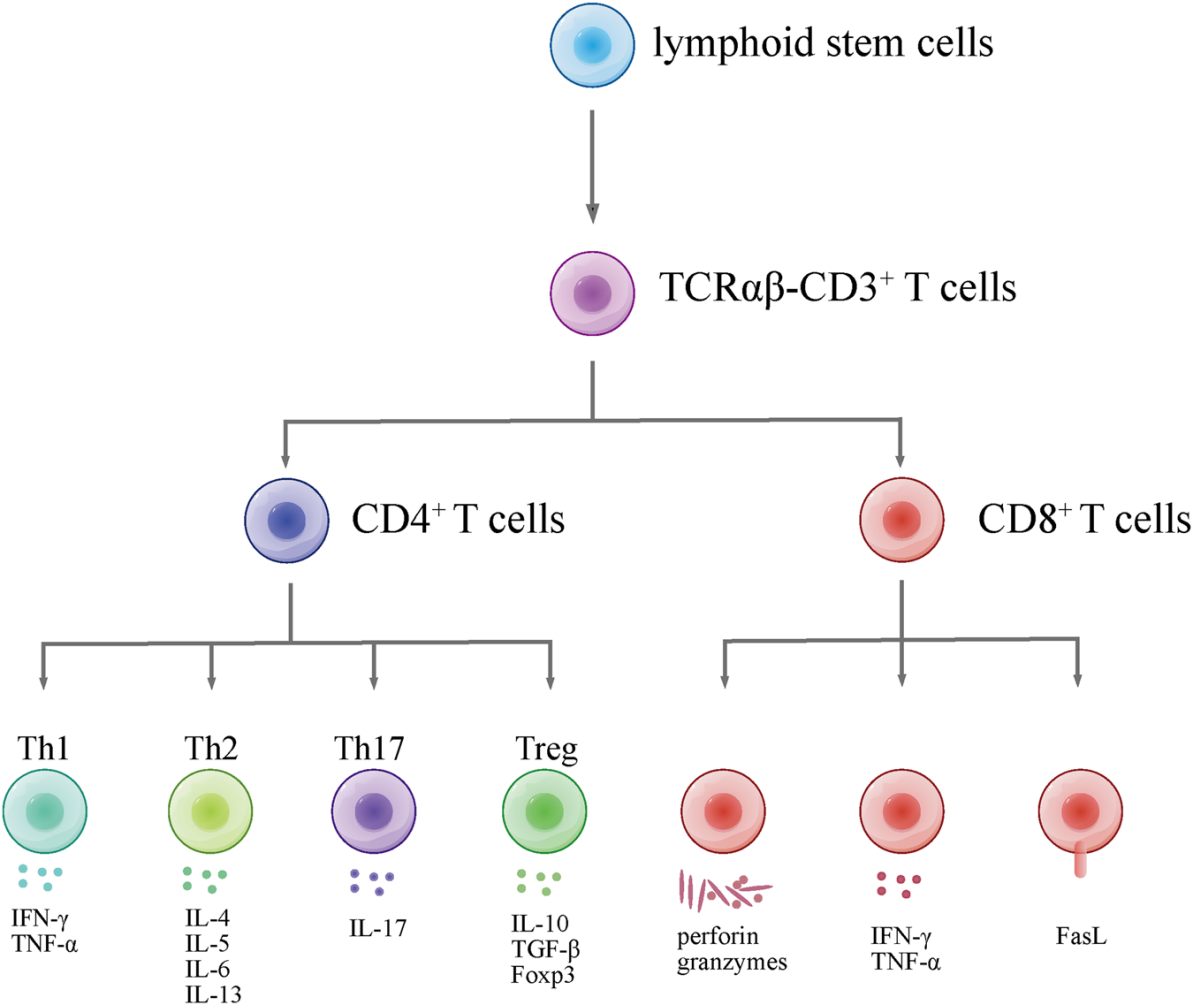
Overview of T cell proliferation and differentiation. Briefly, lymphoid stem cells are originated from stem cells in bone marrow. Then, lymphoid stem cells migrate to thymus, and diverge into CD3^+^ T cells. Subsequently, T cell receptor (TCR) rearrangement occurs within thymus, which results in αβ TCR and γδ TCR. In peripheral blood, TCRαβ CD3^+^ T cells diverge into CD4^+^ and CD8^+^ T cells. CD4+ T cells diverge into Th cells and Treg cells. These cells secret specific cytokines. CD8+ T cell’s function relies on perforin, granzymes, cytokines and FasL.

T cells play a pivotal role in the adaptive immune system, exhibiting a range of fundamental functions. Their ability to recognize antigens, facilitated by distinctive T cell receptors (TCRs), enables the discrimination between self and non-self entities, initiating precise immune responses. Following antigen recognition, T cells undergo activation and clonal expansion, generating effector cells essential for combating specific pathogens. CD3^+^ T cells with αβ TCR undergo differentiation within the thymus, resulting in two primary subsets: CD4^+^ T cells, known as helper T cells, and CD8^+^ T cells, recognized for their cytotoxic activity. CD4^+^ T cells further diversify into specialized types, including Th1, Th2, and Th17 cells, each playing distinct roles in immune responses^20^ (Fig. 2).

Th1 cells play a pivotal role in the immune system by promoting autoimmune diseases through the production of interferon-gamma (IFN-γ). These cells are primarily involved in orchestrating responses against intracellular pathogens and activating macrophages. In contrast, Th2 cells contribute to immune responses by facilitating antibody production. They synthesize interleukins (IL-4, IL-5, IL-6, and IL-13), supporting the maturation and activation of B cells, which, in turn, produce antibodies crucial for combating extracellular pathogens and participating in allergic responses. Th17 cells, on the other hand, are implicated in inflammatory conditions, producing IL-17 and contributing to defense against extracellular bacteria and fungi. The intricate balance between Th1, Th2, and Th17 responses is crucial for maintaining a harmonized immune system. Regulatory T cells (Tregs), characterized by the signature CD4^+^CD25^+^FOXP3^+^ phenotype, play a key role in immune tolerance. They modulate immune responses by secreting cytokines such as IL-10 and TGF-β, contributing to the prevention of excessive immune reactions. Disturbances in the delicate balance between these T cell subsets can lead to the onset or exacerbation of various disorders, including cardiovascular diseases^20–22^.

## T cells in the heart

The development of single-cell/single-nucleus sequencing technology has significantly deepened our understanding of cellular composition within the hearts of both mice and humans^23,24^. In healthy adult mice, immune cells constitute approximately 4.7% of cardiac tissue, comprising macrophages, monocytes, dendritic-like (DC) cells, B cells, T cells, and natural killer (NK) cells^25^. Notably, T/NK cells represent the second largest population of immune cells after monocytes/macrophages. Further analysis by Litviňuková et al. of cardiac cellular composition in healthy human hearts revealed that immune cells make up approximately 8.41% of cardiac tissue^23^. Interestingly, there is a significant discrepancy in immune cell distribution between atrial and ventricular tissues, with immune cells accounting for 10.4% and 5.3% of cells, respectively. This discrepancy suggests a potentially more pronounced role of the immune system in the atria. Moreover, the study identified 21 distinct populations of cardiac immune cells, including 8 subgroups of T cells such as CD4^+^ effector memory T cells, CD4^+^ cytotoxic T cells, CD8^+^ effector memory T cells, and CD8^+^ cytotoxic T cells. Sheng et al. conducted further characterization of CD8^+^ T cells in the left atrium (LA) and found that this specific subgroup exhibits high expression of CCL5, a chemokine that can recruit T cells to infiltrate the heart^26^. Additionally, they observed a significant increase in the number of CD8^+^ and CD4^+^ T cells in the LA of patients with AF compared to those with sinus rhythm. There was also a notable rise in the proportion of infiltrating T/NK cells in the LA of AF patients, exceeding 20% compared to approximately 5% in individuals with sinus rhythm. This comprehensive understanding of cardiac immune cell populations and their distribution sheds light on their potential roles in both health and disease states, particularly in conditions like AF.

## T cells in the AF

Table 1 presents a summary of clinical studies on changes in T cell subtypes in AF. In 2010, activated CD3^+^ T cells were first identified in human left atrial appendages of AF patients using immunofluorescent staining^27^, with higher presence observed in adipose tissue compared to myocardium^28^. Subsequent research has also found a positive correlation between CD3^+^ immunostaining area and the size of the LA, suggesting a link between CD3^+^ T cells and atrial structural remodeling^29,30^. However, there is ongoing debate regarding whether the quantity of CD3^+^ T cells significantly varies among different AF subgroups. Clinical AF can be categorized into paroxysmal (lasting up to 7 days), persistent (lasting longer than 7 days and often requiring treatment to terminate), long-standing persistent (lasting longer than 12 months) and permanent (continuous arrhythmia, and failed or no treatment to restore sinus rhythm)^31^. The number was found to be highest in patients with persistent AF, but lower in those with permanent AF, suggesting that the infiltration of CD3^+^ T cells is influenced by AF duration. Conversely, another study found no statistical difference in CD3^+^ T cell infiltration between paroxysmal and long-standing persistent/permanent AF^30^. However, CD3^+^ T cells in AF patients may exhibit a different and more pro-inflammatory phenotype compared to those in sinus rhythm. Notably, AMPK hyperactivity is known to be crucial for maintaining pro-inflammatory T cells^32,33^. Interestingly, AMPK activity is increased in paroxysmal AF but reduced in long-standing persistent AF patients^34^. Moving forward, adopting more precise quantitative techniques and expanding sample sizes are essential for comprehensive research. Furthermore, in patients with AF, there was an increase in the number of CD3^+^ T cells in peripheral blood, accompanied by elevated expression of CD69 and HLA-DR, which are markers of T cell activation^35^. This highlights the potential role of activated T cells in the pathophysiology of AF. In this section, we briefly introduce evidence of T cell subtype involvement in AF and summarize their potential roles.

**Table 1:** The main studies on the change of T cells in AF

Study	No. of patients	Sample source	Cell types	Findings
Yamashita et al.^27^	11 AF vs. 5 SR	LAA	CD3^+^ T cells, CD8^+^ T cells	Higher infiltration of CD3^+^ T cells in AF patients; some CD3^+^ T cells were CD8^+^, but changes in CD8^+^ T cells were unclear.
Yamashita et al.^29^	21 persistent AF, 6 paroxysmal AF	LAA	CD3^+^ T cells	Positive correlation between LAD and CD3 immunostaining area.
Smorodinova et al.^100^	19 persistent AF vs. 27 SR	LAA, RAA	CD3^+^ T cells	Elevated CD3^+^ T cells in left atrial myocardium instead of right atrial of AF patients.
Hohmann et al.^30^	2 paroxysmal AF vs. 3 persistent AF vs. 3 permanent AF vs. 2 SR	LAA	CD3^+^ T cells	Increased CD3^+^ T cells from SR to paroxysmal to persistent AF; lower in permanent AF than persistent AF.
Wu et al.^28^	20 paroxysmal AF, 30 long-standing persistent/permanent AF	LAA	CD3^+^ T cells	Higher absolute number of CD3^+^ T cells in adipose tissue than myocardium of atria. No difference between AF subtypes.
Chang et al.^35^	45 AF vs. 45 control	Peripheral blood	CD3^+^ T cells, CD4^+^ T cells, CD8^+^ T cells	Higher CD69 and HLA-DR on CD3^+^ T cells in AF; lower PD-1 on CD4^+^ T cells in AF. No difference in PD-1 on CD8^+^ T cells.
Stone et al.^37^	10 permanent AF vs. 20 SR	Public domain micro-array samples from RAA	CD4^+^ T cells, γδ T cells, Treg cells	Association between permanent AF and increased CD4^+^ T cells and γδ T cells. Potential Treg/autoimmune phenotype related to structural remodeling.
Infante et al.^38^	10 AF vs. 11 SR	Peripheral blood	CD4^+^ T cells	Hypomethylated of *CDK5R1, GSE1, HSPG2* and *WDFY3* in AF. Overexpression gene level of *CDK5R1, GSE1, HSPG2* and *WDFY3* in AF.
Sulzgruber et al.^47^	56 CHF-AF vs. 56 CHF	Peripheral blood	CD4^+^CD28^null^ T cells	Higher fraction of CD4^+^CD28^null^ T cells in CHF-AF; associated with cardiovascular mortality and predictive of outcomes in CHF patients with AF.
Sulzgruber et al.^48^	60 POAF vs. 69 non-POAF	Peripheral blood	CD4^+^CD28^null^ T cells	Higher CD4^+^CD28^null^ T cells in POAF; strong predictor for POAF after cardiac surgery, better than NT-proBNP.
Floyd et al.^49^	1137 new on-set AF	Peripheral blood	CD4^+^ T cells, CD8^+^ T cells, Treg cells	No relationship between immune cells (CD4^+^, CD8^+^, Treg) and new-onset AF.
Wu et al.^69^	168 AF vs. 168 control	Peripheral blood	Th17 cells	Higher Th17-related cytokines in AF than control. Positive correlation between Th17-related cytokines and LAD among AF. Negative correlation between Th17-related cytokines and LVEF among AF.
He et al.^71^	25 POAF vs. 63 non-POAF	Peripheral blood	Th17 cells, Treg cells	Higher Th17/Treg in POAF; correlated with CRP level, LA volume, and risk scores. Th17/Treg ratio combined with CRP level is a valuable predictor for POAF.
Wang et al.^70^	40 Rheumatoid Arthritis-AF patients vs. 120 Rheumatoid Arthritis control patients	Peripheral blood	Th1 cells, Th17 cells, Treg cells	Higher Th1, Th1/Th17 ratio, and absolute numbers of Th1 and Th17 cells in RA-AF.
Dai et al.^101^	45 paroxysmal AF vs. 45 chronic AF vs. 45 control	Peripheral blood	Th17 cells, Tim-3^+^ cells	Significant increase in Th17 cells and related cytokines in peripheral blood of AF patients, while Tim-3+ cells and related cytokines were significantly decreased. Higher Th17/Tim-3^+^ cell ratio in chronic AF than paroxysmal AF.
Haemers et al.^94^	12 AF	LAA	CD8^+^ T cells	High infiltration of CD8^+^ T cells in the transition zone between adipocytes and fibrosis area.
Kazem et al.^95^	60 POAF vs. 69 non-POAF	Peripheral blood	CD8^+^CD28^null^ T cells	Higher proportion of CD8+CD28null T cells in POAF patients, significantly associated with the incidence of POAF.
Friebel et al.^96^	80 first-diagnosed AF vs. 20 control	Peripheral blood	CD8^+^ T cells CD3^+^ T cells	Higher activated CD8^+^CD57^+^ T cells in first-diagnosed AF; associated with atrial myopathy and cardiac remodeling. PAR1 activation enhances CD8^+^ T cell effector function and a higher expression of PAR1 in CD8^+^ T cells is linked to increased major adverse cardiovascular events in first-diagnosed AF. Higher CD3^+^PAR1^+^ T cells in FDAF.
Sheng et al.^26^	6 AF vs. 6 SR	LAA	CD8^+^ T cells, CD4^+^ T cells	Increased CD4^+^ and CD8^+^ T cells in AF. CD8^+^ T cells were localized in myocardium and epicardial adipose tissue in AF patients.

*AF* atrial fibrillation, *SR* sinus rhythm, *LAA* left atrial appendage, *RAA* right atrial appendage, *CHF* chronic heart failure, *POAF* post operative atrial fibrillation.

### CD4^+^

CD4^+^ T cells play a pivotal role in regulating adaptive immune responses, capable of differentiating into various Th cell or Treg cell subsets. These subsets can either enhance or suppress the activity of other immune cells, exert direct pro-inflammatory or anti-inflammatory effects on tissue cells, aid in B cell production of high-affinity IgG antibodies, or demonstrate cytolytic activity^36^. Several studies have found a significant increase in pro-inflammatory CD4^+^ T cells in the peripheral blood and atrial infiltrates of AF patients, implicating their involvement in the onset and progression of AF^35,37^. The activation and function of these cells are influenced by factors such as metabolism, the immune microenvironment, and epigenetics. Recently, Infante et al. utilized RRBS (reduced representation bisulfite sequencing) technology to analyze genome-wide CpG methylation patterns in peripheral blood CD4^+^ T cells from patients with AF compared to healthy individuals. They observed increased methylation levels in AF patients, along with reduced methylation and significant upregulation of key genes like *CDK5R1*, *GSE1*, *HSPG2*, and *WDFY3*, effectively distinguishing AF patients from healthy controls. However, further research is required to understand how these altered methylation patterns impact the activation and function of CD4^+^ T cells^38^.

### CD4^+^CD28^null^ T cells

CD4^+^CD28^null^ T cells, lacking CD28 co-stimulatory molecules, behave quite differently from traditional CD4^+^CD28^+^ T cells. Unlike their CD28^+^ counterparts, CD4^+^CD28^null^ T cells are insensitivity to apoptosis induction and resist to the suppressive effects of regulatory T-cells, leading to widespread inflammation and autoimmune reactions^39,40^. Moreover, the absence of CD28 transforms these T cells into cytotoxic entities resembling NK cells, exhibiting cell lytic activities and secreting perforins that harm vascular muscle cells and myocardial tissues^41,42^. Additionally, CD28^null^ cells, compared with CD4^+^CD28^+^ cells, show increased secretion of pro-inflammatory cytokines, including TNF-α and IFN-γ^40,43^.

CD4^+^CD28^null^ T cells, which increase with age^44^ and are elevated in various pathological situations, such as virus infections (CMV, HCV, HIV)^45^, autoimmune disorders, and cardiovascular diseases, are particularly notable in acute coronary syndrome (ACS)^39^. In ACS patients, CD4^+^CD28^null^ T cell expansion correlates with inflammation, condition severity, and recurrence likelihood. Their expansion strongly links to both ACS and coronary events^46^.

Recent studies have highlighted the role of CD4^+^CD28^null^ T cells in AF and cardiovascular outcomes. A 2017 study of congestive heart failure patients found that those with AF had higher levels of CD4^+^CD28^null^ cells, which were predictive of mortality^47^. Similarly, a 2018 investigation linked these cells to perioperative AF (POAF) in cardiac surgery patients^48^. However, a 2023 study involving large cohorts did not find significant associations between CD4^+^CD28^null^ cells and new-onset AF^49^ or ACS^50^. These clinical studies focused on circulating immune cell levels and have limitations, such as using frozen samples and single-time-point assessments. Further research is needed, particularly investigating CD4^+^CD28^null^ cells within atrial tissues of AF patients and in animal models, to better understand their role in AF pathogenesis.

**Fig. 3:**
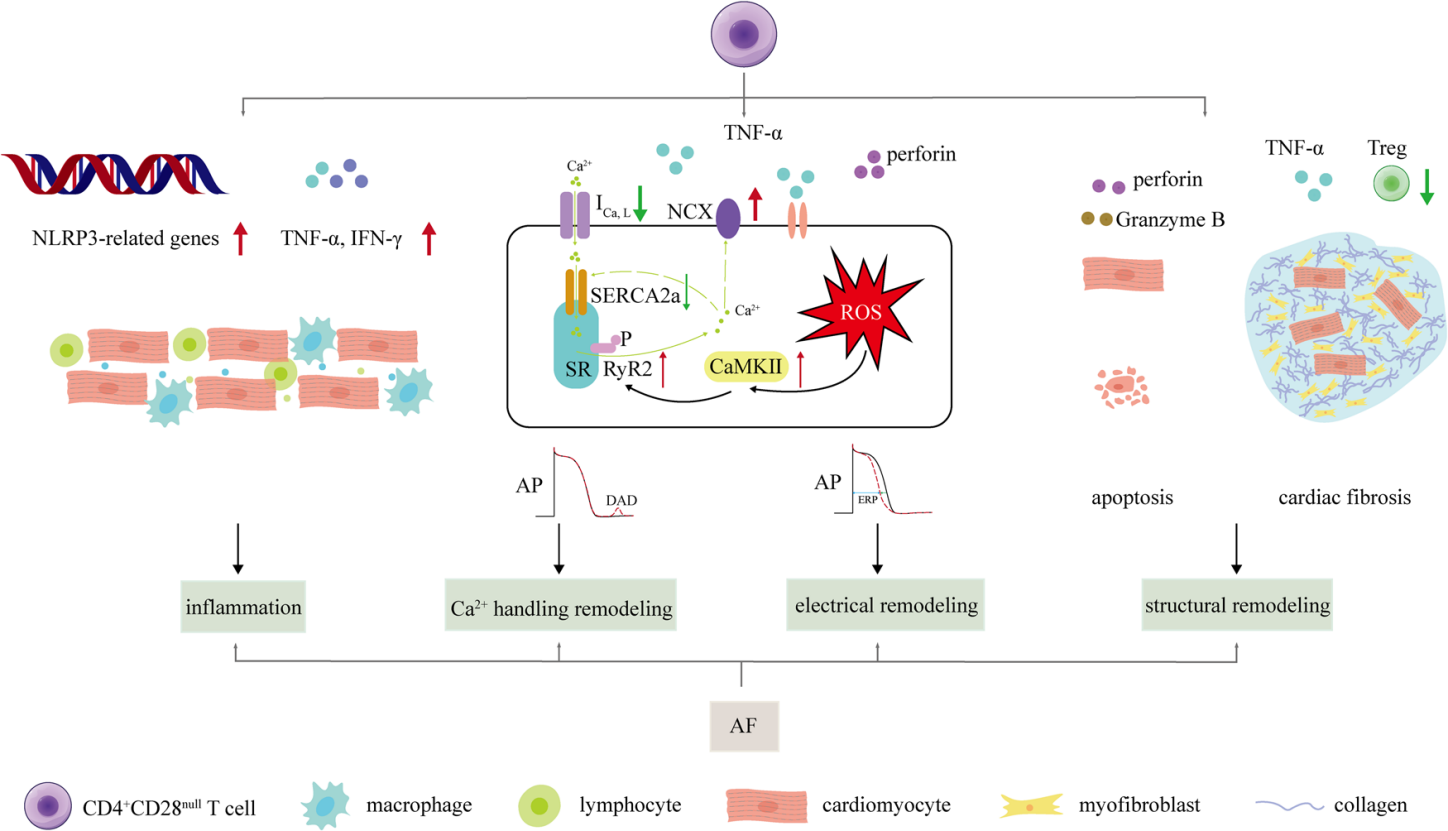
Overview of CD4^+^CD28^null^ cells’ role of AF development. Briefly, the distribution to AF of CD4^+^CD28^null^ cells is including inflammation, Ca^2+^ handling abnormities, electrical remodeling and structural remodeling. CD4^+^CD28^null^ cells secret TNF-α and IFN-γ, along with overexpression of NLRP3-related genes, which promotes cardiac inflammation. Secondly, TNF-α promotes ROS, then promotes Ca^2+^ handling abnormities and electrical remodeling through inhibiting I_Ca,L_ suppressing SERCA2a, enhancing CaMKII, increasing RyR2 expression and phosphorylation and promoting NCX. Perforin also participates these processes. In addition, secretion of perforin facilitates direct damage of Granzyme B, promoting cardiomyocyte apoptosis. CD4^+^CD28^null^ cells’ less responsive to Treg along with TNF-α enhances cardiac fibrosis. Apoptosis and cardiac fibrosis promotes structural remodeling.

CD4^+^CD28^null^ T cells appear to contribute to the pathophysiological processes of AF through several intricate mechanisms (Fig. 3).

Firstly, these cells play a crucial role in atrial inflammation by secreting TNF-α and IFN-γ, which activate not only CD4^+^CD28^null^ cells but also other inflammatory cells like macrophages and neutrophils^51^. Furthermore, CD4^+^CD28^null^ T cells exhibit heightened expression of NLRP3 inflammasome-related genes compared to CD4^+^CD28^+^ T cells, indicating a state of pre-activated inflammation^52^. Activation of NLRP3 plays a crucial role in the development and progression of AF^53^.

TNF-α specifically disrupts calcium homeostasis in atrial myocytes, evidenced by in vitro experiments. In mouse atrial cells, TNF-α increases spontaneous Ca^2+^ release, reduces Ca^2+^ transient amplitudes, and prolongs decay times. These effects are driven by elevated reactive oxygen species (ROS) and CaMKII activation, leading to RyR2 phosphorylation^54^. Kao et al. demonstrated in HL-1 cells that TNF-α suppresses SERCA2a gene expression via promoter hypermethylation, resulting in decreased SERCA2a levels and impaired calcium handling^55^. In rabbit pulmonary vein sleeve cardiomyocytes, TNF-α inhibits *I*_Ca,L_, raising diastolic calcium and enhancing NCX, promoting delayed afterdepolarizations (DADs) and abnormal electrical activity^56^. Studies in mice support these findings, showing that TNF-α deletion or inhibition with agents like etanercept prevents adverse atrial remodeling and exercise-induced AF. TNF-α’s arrhythmogenic effects are associated with disturbed calcium handling, including increased triggered Ca^2+^ release^57,58^. Additionally, TNF-α can induce atrial electrical and structural remodeling. For detailed mechanisms, please refer to relevant reviews on this topic^59^.

Secondly, CD4^+^CD28^null^ T cells are implicated in directly damaging atrial myocytes, affecting their electrical activity and disrupting cardiac electrophysiology, thereby increasing susceptibility to AF. Unlike CD4^+^CD28^+^ cells, CD4^+^CD28^null^ cells exhibit a more pronounced release of perforin, facilitating the entry of toxic proteins like Granzyme B into target cells, ultimately triggering apoptosis^60^. Additionally, exposure of ventricular myocytes to lysed granules or purified perforin induces rapid electrophysiological changes. Investigations have revealed that this exposure can lead to [Ca^2+^]_i_ overload in cardiac myocytes, inducing the generation of a non-selective channel current^61^. However, limited research evidence exists regarding atrial myocytes.

Thirdly, the increased prevalence of the CD4^+^CD28^null^ subset may be associated with the activation of autoimmune responses and subsequent cytotoxicity against atrial structures. Loss of CD28 on these cells reduces responsiveness to the immunoregulatory effects of Treg, making the body more susceptible to aberrant immune reactions. This heightened susceptibility can lead to damage and fibrosis in atrial tissues, exacerbating the onset and progression of AF^39^.

### Th17/Treg

Th17 and Treg cells, essential T cell subsets, maintain immune balance and respond to processes like infections, crucial for immune system function^62^. Th17 cells are pivotal in driving inflammation and fibrosis during cardiac injury, producing key cytokines IL-17 and IL-22, promoting tissue inflammation and adaptive myocardial remodeling^63^. Conversely, Treg cells regulate immune responses, suppressing inflammation and autoimmunity through cytokines like IL-10 and TGF-β, aiding in cardiac tissue healing^64^. The imbalance of Th17/Treg lymphocytes contributes to cardiovascular diseases, including atherosclerosis, angiotensin II-induced ventricular remodeling, and post-myocardial infarction cardiac remodeling^65–67^. Recent advancements in technologies like single-cell sequencing reveal high heterogeneity and plasticity in Th17 and Treg cells, with their functions influenced by the local microenvironment^68^. As our understanding deepens, it’s clear their interplay is integral to immune responses, especially in cardiovascular diseases.

In recent years, several clinical studies have delved into the roles of Th17, Treg, and the Th17/Treg ratio in AF. A retrospective analysis from 2016 proposed that the overactivation of Th17 cells might contribute to the pathogenesis of AF^69^. The study involved 336 AF patients and matched controls using propensity score matching. Despite variations in age, statin/aspirin use, and coronary heart disease prevalence, Th17 cell-related cytokines (IL-17A, IL-17F, IL-21, IL-22) were significantly elevated in AF patients, correlating with AF risk and cardiac ultrasound parameters. In another study, a 1:3 case-control investigation explored T cell subset changes in rheumatoid arthritis-associated AF (RA-AF). Flow cytometry in 40 RA-AF patients and 120 RA controls highlighted higher Th1 and Th17 cells, Th1/Treg ratio, and rheumatoid factor values in RA-AF patients. Logistic regression confirmed associations between these T cell subsets and AF risk^70^. Another study aimed to predict POAF after off-pump coronary artery bypass grafting by assessing the Th17/Treg ratio in 88 patients. POAF patients showed increased Th17 cells and Th17/Treg ratio, correlating with left atrial volume, CRP levels, and CHADS2/CHA2DS2-VASc scores. ROC analysis indicated higher predictive efficacy of the Th17/Treg ratio compared to other biomarkers^71^. In conclusion, these studies collectively underscore the pivotal roles of Th17 cells and the Th17/Treg ratio in AF, suggesting their potential contributions to the pathogenesis of AF. However, it is essential to note that the aforementioned research is primarily derived from single-center studies. Consequently, further validation through multicenter trials and larger sample sizes is imperative to solidify these observations.

The pro-inflammatory and fibrotic effects associated with Th17 cells are predominantly mediated by IL-17. In our study using a rat model of sterile pericarditis (SP), we induced AF through esophageal burst pacing^72,73^. SP rats showed shortened refractory periods, higher AF incidence, and increased AF susceptibility, with elevated atrial inflammation and fibrosis correlating with IL-17A levels. Treatment with anti-IL-17A reduced IL-17A, significantly inhibiting AF and alleviating inflammation and fibrosis^74^. In another AF model induced by acetylcholine and calcium chloride, genomic and bioinformatics analyses demonstrated increased expression of IL-17 in atrial tissues. Curcumin, a natural compound, mitigated fibrosis and reduced AF episodes by inhibiting the IL-17 signaling pathway^75^. Furthermore, Th17 cells were found to activate neutrophils through IL-17A/F, resulting in neutrophil infiltration, myeloperoxidase and ROS production, and conversion of pro-matrix metalloproteinases (pro-MMPs) to MMPs, leading to extracellular matrix degradation and myocardial fibrosis^76^. In SP model rats, anti-IL-17A monoclonal antibody treatment decreased MMP-2 and MMP-9 activity in atrial tissues, along with increased TIMP-2 and TIMP-3 activity^74^. Additionally, prior studies demonstrated the implication of IL-17 in ventricular remodeling post-myocardial infarction in mice^77^, a phenomenon observed in a rabbit myocardial infarction model^78^. These effects may be associated with the enhanced activation of MAPK signaling pathway by IL-17. Furthermore, their research uncovered a critical role for IL-17 in ischemic heart failure models during the generation of ventricular arrhythmias (VAs). IL-17, administered at concentrations of 5, 10, and 20 ng/mL, exhibited a concentration-dependent reduction in conduction velocity, prolonged action potential duration, and increased inducibility of VAs. Knockout of interleukin-17A diminishes VAs susceptibility in diabetic mice^79^. However, confirmation of whether IL-17A affects the action potential and conduction velocity of atrial myocytes requires additional research. These collective findings highlight the intricate involvement of IL-17A in AF pathogenesis and suggest potential therapeutic avenues for managing this cardiac arrhythmia.

Treg cells modulate immune responses by consuming crucial chemotactic and growth factors needed for activated T cells, reducing inflammatory cell aggregation and pro-inflammatory cytokine secretion. They also enhance anti-inflammatory cytokines like IL-10, which protects against cardiac injury^62^. Systemic IL-10 administration suppressed myocardial inflammation and fibrosis, alleviating cardiac remodeling in mouse models of myocardial infarction and pressure overload-induced cardiac hypertrophy, possibly through STAT3 activation^80,81^. Additionally, it may attenuate high-glucose-induced sinoatrial node dysfunction^82^. Recent research highlights the impact of IL-10 deficiency, exacerbating atrial inflammation, fibrosis, and AF induced by a high-fat diet^83^. Splenectomy also intensifies atrial inflammation, fibrosis, and AF induced by pressure overload. Systemic IL-10 administration significantly mitigates these pathological conditions^84^. In a recent study with aged rats, treatment involving B. fragilis administration improved atrial inflammation and fibrosis, reduced AF inducibility, and concurrently increased Treg cell numbers and IL-10 expression in the peripheral blood and spleen. These findings suggest that Treg cells may alleviate atrial inflammation and fibrosis, potentially reducing AF through IL-10 secretion^85^. However, a recent clinical study on non-valvular AF patients revealed an elevated level of IL-10 in peripheral blood, which was correlated with AF episodes^86^. Interestingly, various other cell types, including monocytes-macrophages and even Th17 cells, are capable of secreting IL-10. This prompts intriguing questions about the dynamic changes in the quantity and functionality of Treg cells in the context of AF, as well as their specific mechanistic roles in this setting.

### CD8^+^ T cells

CD8+ T cells, or cytotoxic T lymphocytes, are key players in adaptive immunity against intracellular pathogens and cancer. They recognize and eliminate infected or mutated cells by secreting cytotoxic proteins like perforin and granzymes, or through Fas ligand interactions inducing cell death. They also release pro-inflammatory cytokines such as TNF-α and IFN-γ. Most effector CD8^+^ T cells undergo apoptosis post-response, but 5–10% become memory cells. These memory CD8^+^ T cells quickly respond upon re-exposure to the antigen, efficiently targeting virally-infected and cancer cells. The CD8^+^ T-cell population is diverse, comprising various subsets, including cytotoxic and suppressor T cells^20,87^. The overall impact of CD8^+^ T cells is determined by the dominance of specific subsets, such as CD8^+^CD57^+^ T cells^87^.

CD8^+^ T cells emerge as key contributors to cardiovascular disease pathogenesis, notably in atherosclerosis, as extensively detailed in recent reviews^22,88^. Recent studies using single-cell sequencing reveal increased clonal expansion of CD8^+^ effector memory T cells (low CCR7 and L selectin expression) in plaques, specific to influenza and SARS-CoV-2, and cross-reacting with self-antigens in vascular cells, suggesting autoimmune contributions to atherosclerosis^89^. Regulatory CD8^+^CD25^+^ T cells, with immunosuppressive functions, counteract this process^90^. Autoreactive CD8^+^ T cells also contribute to myocardial damage in immune checkpoint inhibitor (ICI)-associated myocarditis^91^ and autoimmune myocarditis^92^. CD8^+^ T cells are involved in MI, where their depletion post-MI reduces inflammation and preserves ventricular function, a result seen in mice lacking granzyme B^93^. Elevated peripheral blood granzyme B in acute MI patients correlates with higher 1-year mortality risk^93^. Thus, cytotoxic CD8^+^ T cells may contribute to self-tissue damage through the heightened expression of cytotoxic effector molecules such as granzyme B.

In the context of AF, several studies have reported an increase in the number of CD8^+^ T cells in the atria and peripheral blood of patients. Haemers et al. found that patients with permanent AF displayed greater fibrosis and fibro-fatty infiltration in the epicardium of the right atrium compared to those with paroxysmal AF and non-AF patients. Immunohistochemical analysis revealed a focal aggregation of inflammatory cells, predominantly CD8^+^ T cells, at the transition zone between adipose and fibrous tissues, suggesting their involvement in the inflammatory response and fibrotic remodeling in permanent AF^94^. Supporting this, Sheng et al. demonstrated a nearly threefold increase in CD8^+^ T cells in the left atrial appendage of permanent AF patients compared to those in sinus rhythm. These cells infiltrated both the subepicardial fat tissue and the myocardium. Gene expression analysis of these CD8^+^ T cells revealed upregulation of genes associated with T cell activation, oxidative stress response, and leukocyte-cell adhesion^26^.

Kazem et al. investigated the relationship between POAF and CD8^+^ T cells in 129 patients undergoing elective heart valve or coronary artery bypass grafting surgery. They found an increased proportion of peripheral blood CD8^+^ T cells and a higher ratio of CD8^+^CD28^null^/CD8^+^ T cells in the POAF group compared to the non-POAF group. Multivariate regression analysis indicated that the frequency of CD8^+^CD28^null^ T cells was independently associated with POAF occurrence, highlighting their potential predictive value^95^.

Friebel et al. studied 210 patients with first-diagnosis AF (FDAF) and observed an increased proportion of peripheral blood CD8^+^ T lymphocytes expressing the activation marker HLA-DR. Additionally, there was a higher percentage of mature CD8^+^CD57^+^ cells and elevated levels of plasma cytotoxic effector molecules (granulysin, granzymes, sFasL). They also found a close association between the PAR1 signaling pathway and cytotoxic CD8^+^ T cells in FDAF patients, which correlated with cardiovascular events such as cardiovascular death, recurrent hospitalization for AF, heart failure, transient ischemic attacks, ischemic stroke, and acute coronary syndrome^96^. Although PAR4 is typically not expressed on CD8^+^ T cells^97^, its expression in atrial tissue significantly increases under pathological conditions such as obesity and diabetes. This upregulation of PAR4 has been linked to the activation of the NLRP3 inflammasome, potentially contributing to the onset of AF^98^. These findings suggest that PARs signaling may play a role in the potential cross-talk between the innate and acquired immune systems, thereby promoting the onset and progression of AF. However, the detailed mechanisms underlying this interaction remain to be fully elucidated and require further investigation.

The aforementioned clinical research findings suggest a correlation between CD8^+^ T cells and the onset of AF, as well as their association with adverse cardiovascular events. However, these studies predominantly take an observational approach, lacking comprehensive mechanistic investigations. It is postulated that CD8^+^ T cells may contribute to the initiation of AF through various mechanisms: (1) secretion of cytotoxic proteins, such as perforin and granzymes, discussed earlier for their potential to induce apoptosis in atrial myocytes and directly impact their action potentials; (2) release of inflammatory factors, including TNF-α; (3) recruitment of other inflammatory cells like macrophages, leading to local inflammation and oxidative stress, thereby fostering atrial remodeling; (4) direct contact, influencing atrial myocyte electrophysiology through Cx43^99^. However, further research is essential to validate these proposed mechanisms.

## Future perspectives

In summary, recent advances in the understanding of T cell involvement in AF have highlighted the complex interplay between the immune system and cardiac tissue. Both CD4^+^ and CD8^+^ T cells are implicated in AF pathophysiology, promoting inflammation, fibrosis, and atrial remodeling. Dysregulation of T cell subsets, including Th17, and Tregs, underscores the importance of immune balance in cardiac health. Th17 cells contribute to increased inflammation and fibrosis, while a reduction in Tregs is associated with heightened atrial inflammation. CD8^+^ T cells also play a role in structural remodeling, suggesting a multifaceted immune involvement in AF. Moreover, the abnormal proliferation of T cell subtypes, such as CD28^null^ T cells characterized by CD28 receptor downregulation, is associated with both the onset and persistence of AF. This unusual expansion of T cells may increase the risk of cardiovascular events following AF, indicating that the immune system’s role extends beyond mere inflammation and fibrosis to influencing the overall cardiovascular prognosis.

Most studies investigating the relationship between T cells and AF have significant limitations. They primarily focus on quantifying the number of various T cell subtypes in blood or heart tissue in relation to AF presence, but these associations do not establish causality. Moreover, AF diagnosis in these studies is often compromised by the lack of continuous rhythm monitoring, potentially leading to an underestimation of the arrhythmia’s true prevalence. The predominance of observational and retrospective study designs further limits the reliability of these findings. Small sample sizes, along with inadequate control of comorbidities and other risk factors, introduce biases that affect the reported associations between T cells and AF. Consequently, the overall quality of evidence from these clinical studies is relatively weak, which should be carefully considered when interpreting the results.

Future research should focus on elucidating the precise mechanisms of T cell involvement in AF and exploring targeted immunomodulatory therapies. Key areas include studying molecular pathways, epigenetic regulation, and developing biomarkers for early diagnosis and personalized treatment. Clinical trials of immunomodulatory agents, such as cytokine inhibitors and adoptive Treg therapy, are crucial for evaluating therapeutic efficacy. Interdisciplinary collaboration and advanced technologies will drive innovative approaches, aiming to restore immune balance and mitigate AF progression.

## Data Availability

No datasets were generated or analysed during the current study.
