# Author Correction: Recent advances in understanding the roles of T cells in atrial fibrillation

**DOI:** 10.1038/s44325-025-00070-w

**Published:** 2025-06-17

**Authors:** Jiu Pu, Yimei Du

**Affiliations:** 1 Department of Cardiology, Union Hospital, Tongji Medical College, Huazhong University of Science and Technology, Wuhan, China; 2 Research Center of Ion Channelopathy, Union Hospital, Tongji Medical College, Huazhong University of Science and Technology, Wuhan, China; 3 Institute of Cardiology, Union Hospital, Tongji Medical College, Huazhong University of Science and Technology, Wuhan, China; 4 Key Lab for Biological Targeted Therapy of Education Ministry and Hubei Province, Union Hospital, Tongji Medical College, Huazhong University of Science and Technology, Wuhan, China; 5 Hubei Provincial Engineering Research Center of Immunological Diagnosis and Therapy for Cardiovascular Diseases, Union Hospital, Tongji Medical College, Huazhong University of Science and Technology, Wuhan, China

**Keywords:** Immunological disorders, Inflammation, Cardiovascular biology

Correction to: *npj Cardiovascular Health* 10.1038/s44325-024-00026-6, published online 30 September 2024

In this article the Competing Interests statement was incorrect and should have been ‘Author Yimei Du is a Guest Editor at *npj Cardiovascular Health*. Yimei Du was not involved in the journal’s review of, or decisions related to, this manuscript.’ The original article has been corrected.

